# The Controllable Ratio of the Polyaniline-Needle-Shaped Manganese Dioxide for the High-Performance Supercapacitor Application

**DOI:** 10.3390/nano13010101

**Published:** 2022-12-25

**Authors:** Shrouq H. Aleithan, Sajid Ali Ansari, Muhamad Yudatama Perdana, Khan Alam, Zakiah Alhashim, Kawther Al-Amer

**Affiliations:** 1 Department of Physics, College of Science, King Faisal University, P.O. Box 400, Al-Ahsa 31982, Saudi Arabia; 2 Department of Physics, College of Science, King Fahd University of Petroleum and Minerals, Dhahran 31261, Saudi Arabia; 3 Interdisciplinary Research Center for Renewable Energy and Power Systems, King Fahd University of Petroleum and Minerals, Dhahran 31261, Saudi Arabia; 4 Department of Chemistry, College of Science, King Faisal University, P.O. Box 380, Al-Ahsa 31982, Saudi Arabia

**Keywords:** charge–discharge, cyclic stability, supercapacitor, Pani-MnO_2_

## Abstract

The nanohybrid development of metal oxide/conducting polymer as an energy storage material is an active research area, because of the device stability, conductive behavior, and easy fabrication. Herein, needle-like MnO_2_ was coupled with polyaniline fabricated through chemical polymerization followed by the hydrothermal process. The characterization results show that MnO_2_/polyaniline exhibited a needle-like morphology. Different characterization techniques such as X-ray diffraction patterns and scanning electron microscopy confirmed the formation of the MnO_2_/polyaniline nanohybrids. The electrochemical performance, including cyclic voltammetry (CV), galvanostatic charge–discharge (GCD), specific capacitance (C_sp_), and cyclic stability, was examined using a three-electrode assembly cell. The optimized electrode displayed a C_sp_ of 522.20 F g^−1^ at a current load of 1.0 A g^−1^ compared with the other electrodes. The developed synergism during MnO_2_/polyaniline fabrication provided enhanced conductive channels and stability during the charge–discharge process.

## 1. Introduction

The electronic device usage escalation, including wearable lightweight electronics, electronic papers, cell phones, and household applications, has forced the development of alternatives to fulfill daily life demands. This motivates research toward the development of lightweight and durable energy devices; among them are batteries and supercapacitors [1,2]. For any device to achieve cyclic stability, lightweight, excellent power density, fast charge–discharge ability, and cheap cost, it has to be assembled with a supercapacitor, which activates research on energy storage electrode materials [3,4]. Supercapacitor design could be traditional, with a high energy density or a modified high-energy storage for wide application [5,6]. For the demand of super energy storage and short-time period performance, an electrochemical supercapacitor is the best choice [1,2,6]. According to the design and energy storage mechanism, supercapacitors can have two different classifications. The first one is a double-layered capacitor followed by the adsorption and desorption of ions during the electrochemical process, while the second is a pseudo capacitor followed by the oxidation-reduction process as the charge–discharge method [6,7,8,9,10]. Moreover, the electrode configuration determines the supercapacitor’s arrangement. For example, if both electrodes are identical, a symmetric capacitor is formed, whereas an asymmetric one is formed by different electrode types [4,9,11,12,13,14]. Since the design of the electrode materials plays a key role in the performance, several nanomaterials, including metal oxides, carbon-based materials, and polymers have been investigated for developing high-performance supercapacitors [15,16,17,18,19,20]. Metal oxide and hydroxide materials have been extensively explored during the past few decades, including nickel oxide, manganese oxide, cobalt oxide, cobalt hydroxide, and nickel hydroxide. MnO_2_ has been used as an electrode material and received extensive attention due to its low cost, easy synthesis, high theoretical capacitance value, and environmentally sustainable behavior, leading to the development of pseudocapacitor devices. However, the poor electrical conductivity, low surface area, and surface reaction of MnO_2_ during the charge–discharge process led to a lower capacitance value. To resolve these problems, a hybrid MnO_2_ with a conductive polymer, graphene, carbon nanotubes, or bimetallic materials has been developed [14,15]. Conductive polymers provide additional pseudocapacitive behavior and structural stability during the charge–discharge process [16,17]. Different conducting polymers, such as polyaniline, polypyrrole, and polythiophene have been extensively used for the incorporation of MnO_2_ to enhance the electrochemical process [16,17]. Among the conductive polymers, polyaniline has received significant attention owing to its easy fabrication, excellent environmental stability, reversible electrical conductivity, and controlled doping–de-doping chemistry during synthesis [17,18,20,21,22,23,24,25]. However, the performance of the MnO_2_/polyaniline nanocomposite depends on the size, shape, and distribution of MnO_2_ inside the polyaniline matrix. Therefore, MnO_2_ contents inside the polyaniline matrix, MnO_2_ morphologies, and MnO_2_ precursors have been studied to resolve the abovementioned issues. For example, Ansari et al. fabricated fibrous polyaniline and manganese oxide nanocomposites using a simple in situ chemical process which delivered a specific capacitance of 525 F/g at a current load of 2 A/g [19]. Similarly, Lei et al. prepared manganese/polyaniline nanocomposites with manganese having nanowire-like morphology and showing a specific capacitance of 256 F/g at 1.0 A/g current load. In the present work, needle-like MnO_2_ coupled with polyaniline was fabricated through chemical polymerization followed by a hydrothermal process. Different characterization techniques, such as X-ray diffraction patterns, and scanning electron microscopy confirmed the formation of the MnO_2_/polyaniline nanohybrids. The electrochemical performance, including cyclic voltammetry (CV), galvanostatic charge–discharge (GCD), specific capacitance (C_sp_), and cyclic stability, was examined using a three-electrode assembly cell. The optimized electrode (PMO-2, polyaniline-manganese oxide) displayed a specific capacitance of 522.20 F/g at a current density of 1.0 A/g compared to the other electrodes (PP, PMO-1, and PMO-3). This high value is caused by synergism developed during the fabrication of MnO_2_/polyaniline hybrids, which provide enhanced conductive channels and stability during charge–discharge methods.

## 2. Materials

Potassium persulphate, hydrochloric acid, ethanol, and potassium permanganate, aniline monomer were obtained from Sigma-Aldrich (St. Louis, MO, USA). The current collector, which is nickel foam with a thickness of 1.6 mm, a porosity of more than 95%, and a surface density of 346.0 g/m^2^, was obtained from MTI Corporation (Richmond, CA, USA).

### 2.1. Needle-Like MnO_2_ Nanostructure Preparation

The needle-like MnO_2_ nanostructures were synthesized by a hydrothermal method in which 0.5 g of KMnO_4_ was added to 100 mL of 1 M HCl solution and stirred for 30 min. The mixture was transferred to an autoclave and kept at 140 °C for 24 h then it was allowed to cool down to room temperature. The resulting material was collected and washed with water and ethanol then it was dried at 80 °C for 12 h. Moreover, from the reaction process point of view, the reaction for the formation of needle-like MnO_2_ used KMnO_4_ and HCl according to the following equation [20]:

KMnO_4_ + H_2_O + HCl→ MnO_2_ + KCl + H_2_O

### 2.2. Synthesis of Needle-like MnO_2_/Polyaniline Nanocomposites

The needle-like MnO_2_/polyaniline nanocomposites were prepared by in situ oxidative polymerization of aniline monomer, potassium persulfate, and needle-like MnO_2_ that were prepared earlier. In a typical synthesis procedure, as-prepared MnO_2_ was mixed with different moles of aniline monomer using a stirrer and sonication. The aniline adsorbed over the MnO_2_ surface during the stirring and sonication process then freshly prepared potassium persulphate was added dropwise to start the polymerization of the polyaniline over the MnO_2_ surfaces. The mixing of the oxidant led to a change of the solution’s color to greenish black, and this was further stirred for 12 h. The collected precipitate was washed with excess water, acetone, and methanol to remove the acid, potassium persulphate, and oligomers of polyaniline. The composite materials were de-doped with the 1 M ammonia solution to neutralize the remaining acid, which converted the composites into emeraldine base form. The resultant was again doped with the 1 M HCl solution to make it a conductive material. Several samples were synthesized using different amounts of aniline and they are abbreviated as PP (Pure Polyaniline), PMO-1 (9 mL), PMO-2 (5 mL), and PMO-3 (2 mL).

### 2.3. Characterization

Scanning Electron Microscopy (SEM; JEOL JSM-6610 LV with Oxford Instruments X-Max^N^) was conducted to examine the morphologies of the samples. The EDX was further used to analyze elements present in the nanocomposites using AZ tec-22164 software. X-ray diffraction (XRD) of the samples was performed to determine the crystallinity, phase, lattice parameters, and grain size. Vesta software was used to create crystal models for the MnO_2_. Thermogravimetric Analysis (TGA) was employed to examine the thermal properties of the PP, PMO-1, PMO-2, and PMO-3 samples. These studies were carried out using simultaneous DSC/TGA thermal analysis instruments with the SDT Q600 model. The cyclic voltammetry and galvanostatic charge–discharge methods were used to examine the electrochemical properties of PP, PMO-1, PMO-2, and PMO-3 on Nova Auto Lab (Metrohm).

### 2.4. Electrochemical Supercapacitive Performances

Half-cell was assembled to examine the electrochemical performance of PP, PMO-1, PMO-2, and PMO-3 electrode materials. Ag/AgCl was used as reference electrodes, and Pt was used as the counter electrode. The working electrode was prepared by adding active materials to PVDF (10%) and carbon black (10%) to make a slurry-like paste that was used as a coat for nickel foam. The coated nickel foam was further dried in an oven at 60 °C for 12 h. The specific capacitance of all the prepared electrodes of PP, PMO-1, PMO-2, and PMO-3 was calculated using the following equation [26,27]:

C = Idt/mdV(1)

E = 1/2 CV^2^ (2)

P = E/t(3)

where C is the specific capacitance, t is the discharge time, I is current, m is the mass loading of the materials, and V is the applied potential window.

## 3. Results and discussion

**Figure 1:**
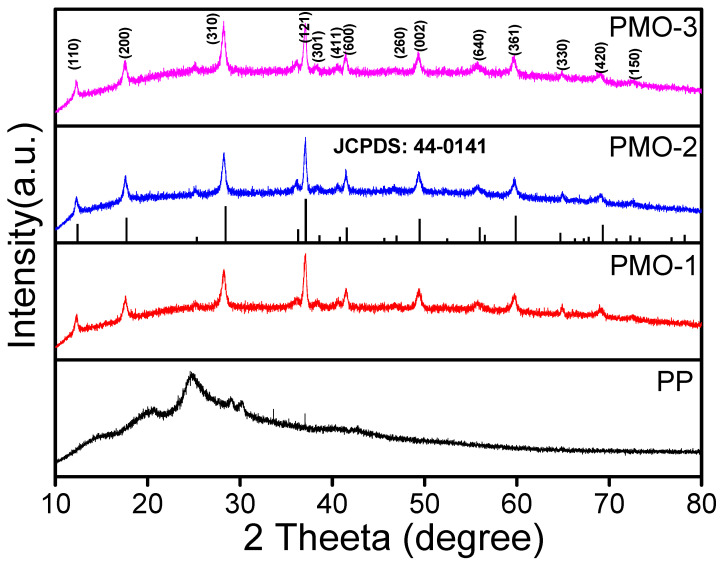
XRD patterns for PP, PMO-1, PMO-2, and PMO-3.

Figure 1 shows the XRD patterns for the PP, PMO-1, PMO-2, and PMO-3 recorded over 2*θ* range from 10° to 80°, in which PP displays broad peaks at 2*θ* = 19.26° and 25.52° along with other accompanying peaks which correspond to the characteristic diffraction pattern (200), (020), and (011) of the polyaniline. The other nanocomposites, PMO-1, PMO-2, and PMO-3, displayed diffraction peaks at 2*θ* = 12.31°, 17.66°, 28.54°, 37.07°, 38.31°, 40.63°, 41.56°, 46.88°, 49.23°, 55.85°, 59.69°, 64.81°, 69.3°, and 72.48°. These peaks corresponded to crystallographic planes (110), (200), (310), (121), (301), (411), (600), (260), (002), (640), (361), (330), (420), and (150), respectively. These results show that all nanocomposites belong to the α-MnO_2_ phase. The simulated XRD spectra of the α-phase of MnO_2_ display a tetragonal crystal structure with a space group of I4/m [24]. A redshift combined with an intensity decrease at 25.04° for all the PMO nanocomposites was noticed in the data. Surface coating of MnO_2_ by polyaniline during the polymerization process could be the cause of the redshift [19]. Lattice parameters for the composites were determined to be a = b = 9.823 Å and c = 2.941 Å for PMO-1, a = b = 9.950 Å, and c = 2.880 Å PMO-2 and =b = 9.787 Å and c = 2.913 Å for case the of PMO-3. These lattice parameter results aligned with JCPDS No. 44-0141 (space group 14/m, a = b = 9.784 Å, c = 2.863 Å) for body-centered tetragonal α-MnO_2_ [26]. Scherrer’s formula was used to determine the crystalline size to be 1.73, 2.00, and 1.72 nm, respectively (Table 1).

**Figure 2:**
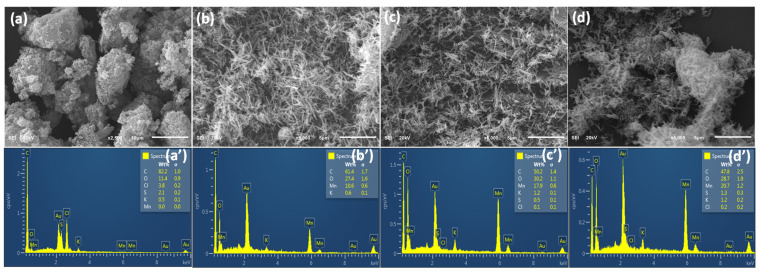
SEM images of (**a**) PP, (**b**) PMO-1, (**c**) PMO-2, and (**d**) PMO-3; EDS spectra of (**a’**) PP, (**b’**) PMO-1, (**c’**) PMO-2, and (**d’**) PMO-3.

SEM images show surface morphologies of PP, PMO-1, PMO-2, and PMO-3 (Figure 2). PP has a nanofiber-like morphology with a large number of interconnected tubules, whereas PMO-1, PMO-2, and PMO-3 display needle-like surface morphologies. The nanofiber structure has an average length larger than 2 μm (Figure 2a). The needle-like structure has diameters of 138.08 nm, 117.11 nm, and 98.41 nm for the PMO-1, PMO-2, and PMO-3, respectively (Figure 2b–d). Figure 2 shows a uniform needle-like morphology related to PMO-2. For PMO-3, MnO_2_ agglomerated on the polyaniline surface because of its excessive amount. Figure 2a’–d’ shows the EDS spectra of PMO-1, PMO-2, and PMO-3, in which large peaks can be seen for Mn, O, and C. The peak for Au is from the conducting tap for the SEM/EDS experiment. Small peaks for S, K, and Cl are also present in the spectrum. For the PP sample, the data show C, S, and Cl peaks only with high intensity compared with the other three samples.

**Figure 3:**
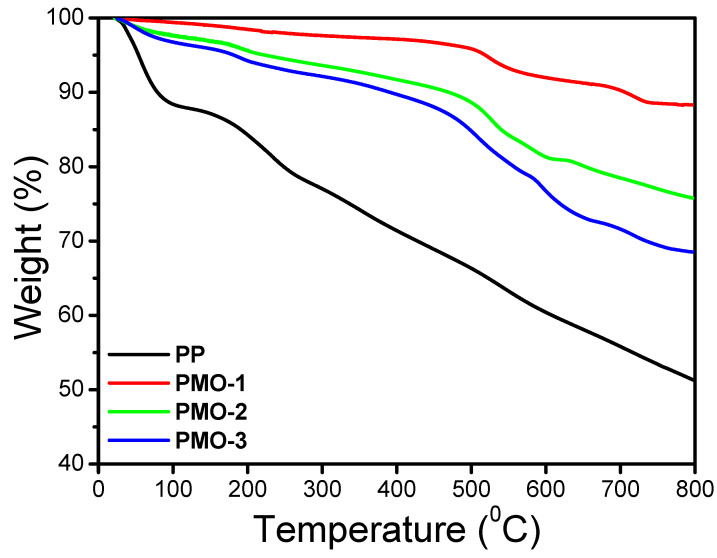
TGA spectra of PP, PMO-1, PMO-2, and PMO-3.

**Figure 4:**
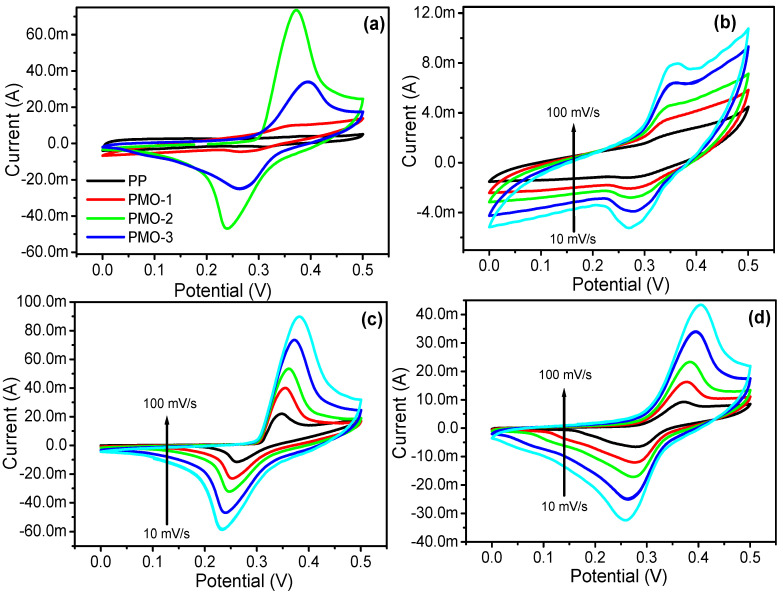
(**a**) Comparative CV graph of PP, PMO-1, PMO-2, and PMO-3 at a fixed scan rate, (**b**) CV graph of PMO-1 at different scan rates, (**c**) CV graph of PMO-2 at different scan rates, and (**d**) CV graph of PMO-3 at different scan rates.

Thermal stability was examined using TGA analysis. All samples were analyzed in the temperature range from 25 to 800 °C (Figure 3). The y-axis represents the weight loss, and the *x*-axis refers to sample temperature. The initial weight loss below 150 °C corresponds to the removal of physio-absorbed water molecules from the samples [18]. Weight loss in the temperature range of 150 to 300 °C is due to the removal of structural water from the α-MnO_2_ phase and deprotonation of PP through the loss of dopant HCl as well as the degradation of small, less stable oligomers in the composite materials [26,27]. For the temperature slightly before 600 °C, weight loss occurs due to the reduction of MnO_2_ to Mn_2_O_3_. This is related to the degradation and decomposition of PP with different polymerization degrees, resulting in the formation of different aliphatic and aromatic fragments at the end, such as p-phenylenediamine, ammonia, N-phenyl-1,4-, carbazole aniline, benzenediamine N-phenylaniline, acetylene pyridine-based heterocycle, and methane, as reported by Ansari et al. [28]. A further reduction in oxygen due to the transition from Mn_2_O_3_ to Mn_3_O_4_ can occur in the temperature range of 700 °C to 850 °C according to the reaction 3Mn_2_O_3_(s) → 2Mn_3_O_4_(s) + ½O_2_ (g) [26]. Figure 4a shows weight loss for the pure polyaniline sample as a linear drop that occurred in two steps. The PMO-1, PMO-2, and PMO-3 nanocomposites have weight losses of 3.92%, 2.19%, and 6.74% that occurred around 390 °C to 570 °C. This was due to the continuous loss of oxygen as MnO_2_ converted to Mn_2_O_3_. Weight losses of 5.36%, 3.04%, and 6.39% occurred in the three samples at around 700 °C to 800 °C, which is related to the loss of oxygen during the phase transformation from Mn_2_O_3_ to Mn_3_O_4_. Comparing the TGA behavior of the PP, and PMO hybrids samples, the PMO samples are more thermally stable as their degradation curves are above that of PP. The thermal stability of the PMO hybrids is directly related to stabilizing effects of MnO_2_ on PP. A similar stabilizing interaction was also reported for composites of PP with CNT and graphene [29].

### Electrochemical Performance in the Half Cell

**Figure 5:**
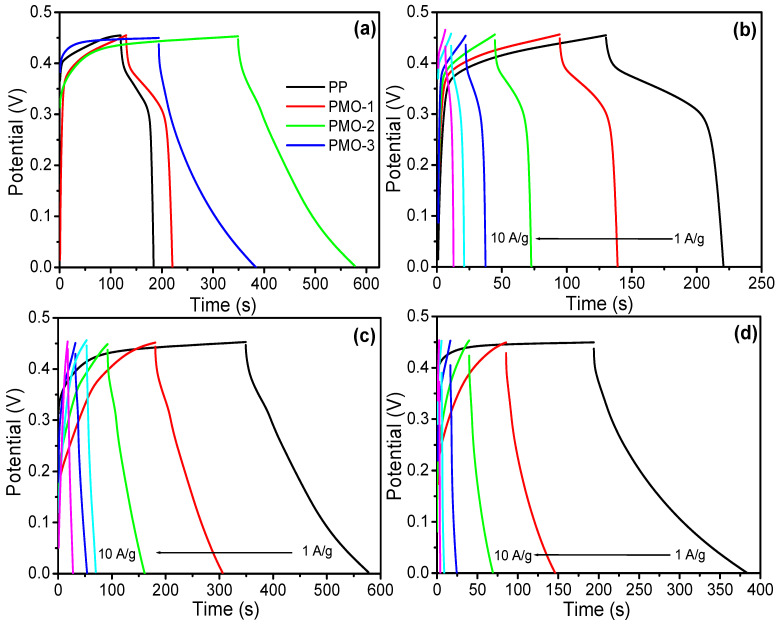
(**a**) Comparative GCD graph of PP, PMO-1, PMO-2, and PMO-3 at a fixed current load, (**b**) GCD graph of PMO-1 at different current densities, (**c**) GCD graph of the PMO-2 at different current densities, and (**d**) GCD graph of the PMO-3 at different current densities.

Aiming to understand the electrochemical supercapacitive behavior and the energy storage performance of all the samples (PP, PMO-1, PMO-2, and PMO-3) that could provide a pathway to the practical application of the electrode materials, the galvanostatic charge–discharge and cyclic voltammetry performance of the electrode were examined in a three-electrode assembly cell within a 3 M KOH electrolytic solution. The CV test was performed in a potential window of 0.0 to 0.5 V at varying scan rates of 10 to 100 mV s^−1^ (Figure 5). The fabricated MnO_2_/polyaniline nanohybrids exhibited exceptional electrochemical performance, which was expected from the SEM results as they show a PMO branch-like network structure. Branch-like structure leads to a shortening of the charges’ diffusion path and a deep redox reaction which simultaneously enhances the overall electrochemical performance of the materials [30,31,32].

The comparative cyclic voltammetry measurement for the nanocomposite electrode material was performed at a scan rate of 50 mV/s, as illustrated in Figure 4a. The results showed that the PMO-2 integrated area is larger than that of PP, PMO-1, and PMO-3, which suggests a synergistic effect between the MnO_2_ and polyaniline in the optimized composite electrode (PMO-2). The redox peak in the CV curves (Figure 4a) shows in PMO-1, PMO-2 and PMO-3, but in the case of PP, the CV is nearly rectangular in shape. The CV measurement of all electrode materials, PMO-1 (Figure 4b), PMO-2 (Figure 4c), and PMO-3 (Figure 4d), was taken at different scan rates, which shifted the redox peak as it increased. The shift in redox peak with increasing scan rates could be explained by highly reversible and fast charge transfer during the electrochemical test, which implies lower polarization of the electrode materials and fast electron and ion transport rates during the electrochemical processes. Based on an electroanalytical point of view, the small potential difference in anodic and cathodic peaks may be attributed to the reversible electrochemical reaction, which can be explained by the above reaction equation [33,34]. The reversibility and integrated capacitive area of the electrode during the faradic reaction were homogeneously maintained (Figure 4b–d), even at a higher scan rate, which supports the existence of increased ion diffusion during the electrochemical procedure. CV curves show that nanostructures conduct electrons and ions at higher scan rates due to a significant increment in the current at higher scan rates [30]. Moreover, the behavior of PMO-1 CV is almost same as that of PP due to the lower ratio of manganese oxide present in this compound. The increased in the amount of aniline monomer in PMO-2 and PMO-3 is mention in the synthesis section, while the increased percentage of MnO_2_ in all electrodes calculated by EDS is mentioned in Figure 2.

To understand the exact charge storage mechanism and potential specific capacitance storage of the fabricated composite electrode materials, the charge–discharge analysis was performed. The GCD profiles of all fabricated electrode materials, PP, PMO-1, PMO-2, and PMO-3, were determined at fixed and different current densities. The corresponding specific capacitance was calculated from the above charge–discharge equation. The GCD SC values of the activated electrode materials were estimated at different current densities ranging from 1 to 10 Ag^−1^. Figure 5a shows a comparative GCD for PP, PMO-1, PMO-2, and PMO-3 at fixed current density, and indicates that PMO-2 has longer charging–discharging behavior compared to other samples, leading to a good specific capacitance. The comparative CV and GCD graphs indicate that the optimized PMO-2 nanohybrid is an appropriate choice for the fabrication of supercapacitor electrode material. At the fixed current density of 1 Ag^−1^, the optimized PMO-2 nanohybrid electrode exhibited a value of 522.2 F g^−1^ with a long discharge time, which is 3.2 times higher than that of PP (162.1 F g^−1^), and 3.2 times higher than that of PMO−1 (198.3 F g^−1^) and PMO−3 (419.8 F g^−1^).

**Figure 6:**
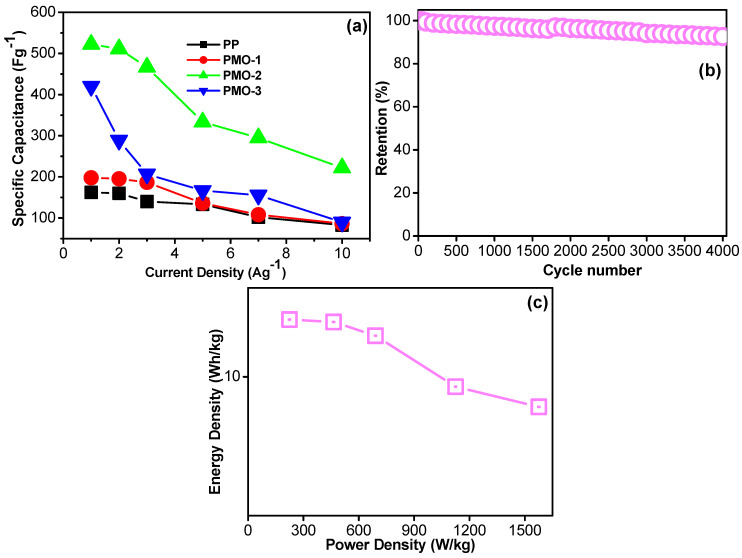
(**a**) Specific capacitance graph of PP, PMO-1, PMO-2, and PMO-3 recorded at a current density of 1–10 A/g; (**b**) cyclic stability graph of the PMO-2 electrode; and (**c**) power and energy density spectra of the PMO-2 electrode.

The elongated charge–discharge duration, large integrated area, and improved specific capacitance of the PMO-2 nanohybrid electrode can be attributed to the synergy that develops due to the presence of optimized amounts of metal and polymer in composite electrode materials [35,36]. This plays an important role in providing a large number of active sites and facilitating excessive ion diffusion during the electrochemical procedure. The specific capacitance of individual electrodes was also measured at different current densities using GCD (Figure 5 and Figure 6a), and the results are shown in Figure 5b–d. The potential range reached the specific level due to its lower conductivity. Some electrode materials had a longer charging time to reach their potential range at lower current density. This type of behavior is generally observed in some transition metal compounds and pseudocapacitors in 3-electrode measurement. If we could test the same materials in a practical system, this erroneous behavior would not appear. The resulting specific capacitance values of the PP, PMO-1, PMO-2, and PMO-3 electrodes were extracted from the discharge curve and equation. At current densities of 1, 2, 3, 5, 7, and 10 Ag^−1^, the specific capacitance of the optimized PMO-2 nanohybrid composite was around 522.2, 511.1, 466.6, 333.3, 295.1, and 222 Fg^−1^, respectively (Figure 5c). For PP, it was around 163, 158, 140, 133, 108, and 88 Fg^−1^, respectively. For the PMO-1, the specific capacitances were around 197.8, 195.5, 186.6, 130.6, 110.5, and 84 Fg^−1^, respectively (Figure 5b). For PMO-3, the calculated specific capacitances were around 420, 288.8, 206.6,166, 154.1, and 92.4 Fg^-1^, respectively (Figure 5d). These results confirm that the PMO-2 nanohybrid has an excellent capacitance performance compared with the other electrodes fabricated in the present work. Table 2 displays the previously reported MnO_2_/polyaniline nanohybrid supercapacitor from the literature compared to the optimized PMO-2 in this work. The measured supercapacitive values and rate capability (%) of the active electrode materials were plotted against varying current densities, as illustrated in Figure 6a and b. The optimized PMO-2 nanohybrid composite delivered a higher capacitance, demonstrating a good rate capability.

The stability of the electrode material is a major issue of concern and one of the most important parameters to consider when investigating potential energy storage applications. In the present case, the consecutive charge–discharge cycle test was conducted, and the results are depicted in Figure 6b. Compared to PP, the optimized PMO-2 nanohybrid composite exhibited excellent performance in the cyclic stability test over 4000 cycles run at a fixed current load. The stability results also show that the specific capacitance was also maintained over all cycles, and retention did not drop quickly, which supports the excellent properties of the fabricated optimized PMO-2 nanohybrid electrode material. However, compared with the PMO-2 nanohybrids, the retention percentage of PP decreased much faster after starting the charge–discharge cycles. This implies that the optimized PMO-2 nanohybrid electrode has good rate capability, long life cycle stability, and superior specific capacitive properties, suggesting that it has great potential for energy storage applications.

Moreover, the two major parameters used for evaluating the practicability of the supercapacitors, namely the energy and power density, were recorded during the electrochemical measurements and further calculated using equations 2 and 3 [37,38,39]. These values related to PMO-2 nanohybrid electrodes were calculated at various current densities, as shown in Figure 6c (Ragone plot). The Ragone plot shows that the energy density decreased when the power density increased, which is in accordance with the calculated specific capacitance value of the electrodes. This behavior can be justified as follows: at a high current load, the charging and discharging procedures are fast, and the electrolyte ions do not have sufficient time to penetrate available pores on the electrode surface. In the present case, the optimized PMO-2 nanohybrid electrodes exhibited the highest energy density of 14.6 Wh/kg at a power density of 224.6 W/kg, demonstrating the excellent high-power performance of the prepared electrode. Moreover, the PMO-2 nanohybrid electrode achieved the highest power density of 1603.3 W/kg, corresponding to an energy density of 4.8 Wh/kg.

## 4. Conclusions

The MnO_2_/polyaniline nanohybrids were developed through a simple hydrothermal process combined with the chemical polymerization method. The needle-like MnO_2_ coupled with polyaniline was further assembled into a three-electrode assembly cell as a working electrode, and its electrochemical supercapacitive performance was assessed using the CV and GCD techniques in an aqueous electrolyte. The optimized electrode (PMO-2) displayed a C_sp_ of 522.20 F g^−1^ at a current load of 1.0 A g^−1^ and exhibited good cyclic stability under various charge–discharge processes. The enhanced performance of the PMO-2 electrode is credited to the combined properties of the individual components of the MnO_2_, conduction polyaniline, and their nanohybrid, which provides enhanced conductive channels and stability due to the synergism developed during the fabrication of the MnO_2_/polyaniline. The excellent performance of the PMO-2 electrode introduces it as a good choice for developing energy devices to serve various applications.

## Figures and Tables

**Table 1:** FWHM and crystalline size of the nanocomposites.

Sample	2*θ*	(hkl)	FWHM (°)	Crystalline Size (nm)
**PMO-1**	37.07	(121)	0.1597	1.73
**PMO-2**	37.19	(121)	0.1382	2.00
**PMO-3**	37.07	(121)	0.1603	1.72

**Table 2:** Comparison of the performance of the present electrode with previous published work.

Electrode	Synthesis	Electrolyte	Specific Capacitance at Current Density	Retention%	Ref.
MnO_2_-PANI	Coating and grafting	1 M H_2_SO_4_	407 F g^−1^ at 0.5 mA cm^−2^	96.4% after 2000 cycles	1
PAni-MnO_2_ composites	Chemical oxidative polymerization	0.5 M Na_2_SO_4_	242 F g^−1^ at 0.1 A g^−1^	99% after 1000 cycles	16
γ-MnO_2_/PANI	In situ polymerization	1 M H_2_SO_4_	232 F g^−1^ at 1 A g^−1^	78.6% after 3000 cycles	17
MnO_2_/PANI	One-step interfacial polymerization	HClO_4_	168 F g^−1^ at 0.2 mA cm^−2^	95% after 1000	4
RGO/MnO_2_/PANI	Electrodeposition and chemical oxidative polymerization	1.0 M Na_2_SO_4_	636.5 F g^−1^ at 1.0 A g^−1^	85% after 10,000 cycles	5
MnO_2_-PANI-GO	Two step coating method	1.0 M Na_2_SO_4_	522 F g^−1^ at 0.25 A g^−1^	97% after 5100 cycles	6
fibrous Pani–MnO_2_	In situ chemical oxidative method	0.5 M H_2_SO_4_	525 F g^−1^ at a current density of 2 A g^−1^	76.9% after 1000 cycles	19
PANI-MnO_2_	Exchange reaction method	0.1 M Na_2_SO_4_	330 F g^−1^ at 1 A g^−1^	94% after 1000 cycles	8
Pani-MnO_2_ nanowire	In situ chemical oxidative method	1 M KOH	256 F g^−1^ at 1 A g^−1^	--	20
MnO_2_/polyaniline nano-hybrids	Hydrothermal and chemical oxidative polymerization	3 M KOH	522 F g^−1^ at 1 A g^−1^	91% after 4000 cycles	This work

## Data Availability

The data presented in this study are available on request from the corresponding author.

## References

[1] Yan J., Fan Z.J., Sun W., Ning G.Q., Wei T., Zhang Q., Zhang R.F., Zhi L.J., Wei F. (2012). Advanced Asymmetric Supercapacitors Based on Ni(OH)_2_/Graphene and Porous Graphene Electrodes with High Energy Density. Adv. Funct. Mater..

[2] Zhang C., Lv W., Tao Y., Yang Q.H. (2015). Towards superior volumetric performance: Design and preparation of novel carbon materials for energy storage. Energy Environ. Sci..

[3] Parveen N., Ansari S.A., Ansari S.G., Fouad H., Abd El-Salam N.M., Cho M.H. (2018). Solid-state symmetrical supercapacitor based on hierarchical flower-like nickel sulfide with shape-controlled morphological evolution. Electrochim. Acta.

[4] Ansari M.Z., Ansari S.A., Kim S.-H. (2022). Fundamentals and recent progress of Sn-based electrode materials for supercapacitors: A comprehensive review. J. Energy Storage.

[5] Parveen N., Ansari S.A., Alamri H.R., Ansari M.O., Khan Z., Cho M.H. (2018). Facile Synthesis of SnS_2_ Nanostructures with Different Morphologies for High-Performance Supercapacitor Applications. ACS Omega.

[6] Parveen N., Ansari M.O., Cho M.H. (2015). Simple route for gram synthesis of less defective few layered graphene and its electrochemical performance. RSC Adv..

[7] Peng H., Ma G., Mu J., Wang H., Lei Z. (2014). High-performance supercapacitor based on multi-structural CuS@polypyrrole composites prepared by in-situ oxidative polymerization. J. Mater. Chem. A.

[8] Wang Y., Xie Y. (2020). Electroactive FeS_2_-modified MoS_2_ nanosheet for high-performance supercapacitor. J. Alloy. Compd..

[9] Miao Y.E., Fan W., Chen D., Liu T. (2013). High-Performance Supercapacitors Based on Hollow Polyaniline Nanofibers by Electrospinning. ACS Appl. Mater. Interfaces.

[10] Zhong S., Sun T., Yuan G., Wen R., Chen W., Wang Z., Zhang L., Zhan K., Zhu M., Yang J. (2022). Scalable fabrication of NiCo_2_O_4_/reduced graphene oxide composites by ultrasonic spray as binder-free electrodes for supercapacitors with ultralong lifetime. J. Mater. Sci. Technol..

[11] Liu Y., Zhou Z., Zhang S., Luo W., Zhang G. (2018). Controllable synthesis of CuS hollow microflowers hierarchical structures for asymmetric supercapacitors. Appl. Surf. Sci..

[12] Xia H., Meng Y.S., Yuan G., Cui C., Lu L. (2012). A Symmetric RuO_2_/RuO_2_ Supercapacitor Operating at 1.6 V by Using a Neutral Aqueous Electrolyte. Electrochem. Solid-State Lett..

[13] Ranganatha S., Munichandraiah N. (2018). γ-MnS nanoparticles anchored reduced graphene oxide: Electrode materials for high performance supercapacitors. J. Sci. Adv. Mater. Devices.

[14] Ren H., Zhang L., Zhang J., Miao T., Yuan R., Chen W., Wang Z., Yang J., Zhao B. (2022). Na^+^ pre-intercalated Na_0.11_MnO_2_ on three-dimensional graphene as cathode for aqueous zinc ion hybrid supercapacitor with high energy density. Carbon.

[15] Huang M., Li F., Dong F., Zhang Y.X., Zhang L. (2015). MnO_2_-based nanostructures for high-performance supercapacitors. J. Mater. Chem. A.

[16] Roy H.S., Islam M.M., Mollah M.Y.A., Susan M.A.B.H. (2020). Polyaniline-MnO_2_ composites prepared in-situ during oxidative polymerization of aniline for supercapacitor applications. Materials.

[17] Zhu Y., Xu H., Tang J., Jiang X., Bao Y., Chen Y. (2021). Synthesis of γ-MnO_2_/PANI Composites for Supercapacitor Application in Acidic Electrolyte. J. Electrochem. Soc..

[18] Parveen N., Ansari M.O., Cho M.H. (2016). Route to High Surface Area, Mesoporosity of Polyaniline–Titanium Dioxide Nanocomposites via One Pot Synthesis for Energy Storage Applications. Ind. Eng. Chem. Res..

[19] Ansari S.A., Parveen N., Han T.H., Ansari M.O., Cho M.H. (2016). Fibrous polyaniline@manganese oxide nanocomposites as supercapacitor electrode materials and cathode catalysts for improved power production in microbial fuel cells. Phys. Chem. Chem. Phys..

[20] Wu J., Huang H., Yu L., Hu J. (2013). Controllable Hydrothermal Synthesis of MnO_2_ Nanostructures. Adv. Mater. Phys. Chem..

[21] Sarma M.P., Wary G. (2016). Synthesis and characterization of chemically deposited nanocrystalline PbS thin film. Adv. Sci. Lett..

[22] Patil A.M., Lokhande A.C., Chodankar N.R., Kumbhar V.S., Lokhande C.D. (2016). Engineered morphologies of β-NiS thin films via anionic exchange process and their supercapacitive performance. Mater. Des..

[23] Gospodinova N., Ivanov D.A., Anokhin D.V., Mihai I., Vidal L., Brun S., Romanova J., Tadjer A. (2009). Unprecedented Route to Ordered Polyaniline: Direct Synthesis of Highly Crystalline Fibrillar Films with Strong p-p Stacking Alignment. Macromol. Rapid Commun..

[24] Elmacı G., Ertürk A.S., Sevim M., Metin Ö. (2019). MnO_2_ nanowires anchored on mesoporous graphitic carbon nitride (MnO_2_@mpg-C_3_N_4_) as a highly efficient electrocatalyst for the oxygen evolution reaction. Int. J. Hydrog. Energy.

[25] Selvakumar K., Kumar S.M.S., Thangamuthu R., Ganesan K., Murugan P., Rajput P., Jha S.N., Bhattacharyya D. (2015). Physiochemical Investigation of Shape-Designed MnO_2_ Nanostructures and Their Influence on Oxygen Reduction Reaction Activity in Alkaline Solution. J. Phys. Chem. C.

[26] Umek P., Korošec R.C., Gloter A., Pirnat U. (2011). The control of the diameter and length of α-MnO_2_ nanorods by regulation of reaction parameters and their thermogravimetric properties. Mater. Res. Bull..

[27] Rana U., Chakrabarti K., Malik S. (2012). Benzene tetracarboxylic acid doped polyaniline nanostructures: Morphological, spectroscopic and electrical characterization. J. Mater. Chem..

[28] Ansari M.O., Mohammad F. (2012). Thermal Stability of HCl-Doped-Polyaniline and TiO_2_ Nanoparticles-Based Nanocomposites. J. Appl. Polym. Sci..

[29] Ansari M.O., Yadav S.K., Cho M.H., Mohammad F. (2013). Thermal stability in terms of DC electrical conductivity retention and the efficacy of mixing technique in the preparation of nanocomposites of graphene/polyaniline over the carbon nanotubes/Polyaniline. Compos. Part B.

[30] Yang J., Duan X., Guo W., Li D., Zhang H., Zheng W. (2014). Electrochemical performances investigation of NiS/rGO composite as electrode material for supercapacitors. Nano Energy.

[31] Jadhav S.K.A., Dhas S.K.D., Patil K.T., Moholkar A.V., Patil P.S. (2021). Polyaniline (PANI)-manganese dioxide (MnO_2_) nanocomposites as efficient electrode materials for supercapacitors. Chem. Phys. Lett..

[32] Meng F., Yan X., Zhu Y., Si P. (2013). Controllable synthesis of MnO_2_/polyaniline nanocomposite and its electrochemical capacitive property. Nanoscale Res. Lett..

[33] Li H., He Y., Pavlinek V., Cheng Q., Sahab P., Li C. (2015). MnO_2_ nanoflake/polyaniline nanorod hybrid nanostructures on graphene paper for high performance flexible supercapacitor electrodes. J. Mater. Chem. A.

[34] Han G., Liu Y., Zhang L., Kan E., Zhang S., Tang J., Tang W. (2014). MnO_2_ Nanorods Intercalating Graphene Oxide/Polyaniline Ternary Composites for Robust High-Performance Supercapacitors. Sci. Rep..

[35] Zhang X., Ji L., Zhang S., Yang W. (2007). Synthesis of a novel polyaniline-intercalated layered manganese oxide nanocomposite as electrode material for electrochemical capacitor. J. Power Sources.

[36] Chen L., Song Z., Liu G., Qiu J., Yu C., Qin J., Ma L., Tian F., Liu W. (2013). Synthesis and electrochemical performance of polyaniline–MnO_2_ nanowire composites for supercapacitors. J. Phys. Chem. Solids.

[37] Zhao X., He D., You B. (2022). Laser engraving and punching of graphene films as flexible all-solid-state planar micro-supercapacitor electrodes. Mater. Today Sustain..

[38] Zheng C., Zhang J., Zhang Q., You B., Chen G. (2015). Three dimensional Ni foam-supported graphene oxide for binder-free pseudocapacitor. Electrochim. Acta.

[39] You B., Li N., Zhu H., Zhu X., Yang J. (2013). Graphene Oxide-Dispersed Pristine CNTs Support for MnO_2_ Nanorods as High Performance Supercapacitor Electrodes. ChemSusChem.

